# Substance use disorders and avoidable mortality after prison

**DOI:** 10.1016/S2215-0366(15)00125-X

**Published:** 2015-04-21

**Authors:** Sarah E Wakeman, Josiah D Rich

**Affiliations:** Massachusetts General Hospital Substance Use Disorder Initiative, Harvard Medical School, Boston, MA, USA (SEW); and The Center for Prisoner Health and Human Rights at the Miriam Hospital, Brown University, Providence, RI 02906, USA

Worldwide, more than 30 million people spend time in prison every year.^1^ The USA incarcerates 25% of these people and one in 31 Americans is currently under correctional control, either in jail, prison, or on probation or parole.^2^ Most prisoners will eventually be released, and the 2 weeks after release have been shown to be associated with a substantial increase in mortality, especially from overdose.^3^ Substance use disorders are highly prevalent among incarcerated populations, with more than half of prisoners in some countries being imprisoned for drug-related convictions.^4^ In the USA, 85% of people in prisons or jails are substance involved, with 1·5 million individuals meeting DSM criteria for a substance use disorder and an additional 458 000 either with a history of substance use, under the influence at the time of arrest, or convicted of a crime committed to obtain money to buy drugs.^5^

Addiction is a treatable disease and decades of scientific evidence support the efficacy of treatment to improve clinical outcomes, save lives, and reduce societal costs. Treatment for opioid use disorder during incarceration with agonists such as buprenorphine or methadone has been shown to reduce recidivism, improve treatment retention, reduce illicit drug use, and decrease criminal activity.^6,7^ Buprenorphine has also been shown to decrease the risk of overdose death by more than 50%.^8^ However, despite the overwhelming evidence, treatment remains variable between correctional facilities and few prisoners receive these life-saving drugs.^9^

In *The Lancet Psychiatry*, Zheng Chang and colleagues^10^ examined mortality in all people released from prison in Sweden between Jan 1, 2000, and Dec 31, 2009. In this sample of 47 326 individuals and 238 457 person-years of follow-up, the researchers reported that substance use (both alcohol and illicit drug use) was related to a substantial proportion of post-release mortality, even when controlling for other factors using imprisoned siblings as controls. The association between mental illness and post-release mortality disappeared when substance use was controlled for. This well designed study of an entire country offers important and concerning new data on the high risk of death for individuals with substance use disorder who are incarcerated. The results of the study also showed that the period of risk of increased mortality after release from prison is much longer— months to years—than the few weeks previously reported,^3^ an important finding that is probably true in most places. These findings are even more alarming when considering the magnitude of risk for a country such as the USA, which has a much higher incarceration rate and far more drug-related convictions than does Sweden.

Access to effective treatments for addiction, particularly pharmacotherapy, is the single greatest intervention that can reduce the death toll from overdose.^11^ The withholding of evidence-based treatment for prisoners is arguably unethical and certainly unwise. In the USA, correctional facilities are mandated by the Supreme Court to provide medical care that meets the community standard.^12^ And yet, within state prisons people with drug use disorders largely go without care: of these people, only 0·8% receive detoxification services, 0·3% receive maintenance pharmacotherapy, 6·5% receive counselling by a professional, and 9·5% receive treatment in a residential facility.^13^ Even those on treatment in the community are systematically forced off when incarcerated, with detrimental consequences.^14^ The absence of care in this deeply affected population translates into high costs to society and the communities that these individuals return to. As the Article shows, these costs also translate into avoidable deaths from a treatable illness.
